# Twenty amino acids at the C-terminus of PA-X are associated with increased influenza A virus replication and pathogenicity

**DOI:** 10.1099/vir.0.000143

**Published:** 2015-08

**Authors:** Huijie Gao, Honglei Sun, Jiao Hu, Lu Qi, Jinliang Wang, Xin Xiong, Yu Wang, Qiming He, Yang Lin, Weili Kong, Lai-Giea Seng, Juan Pu, Kin-Chow Chang, Xiufan Liu, Jinhua Liu, Yipeng Sun

**Affiliations:** ^1^​ Key Laboratory of Animal Epidemiology and Zoonosis, Ministry of Agriculture, College of Veterinary Medicine and State Key Laboratory of Agrobiotechnology, China Agricultural University, Beijing, PR China; ^2^​ Animal Infectious Disease Laboratory, School of Veterinary Medicine, Yangzhou University, Yangzhou, Jiangsu Province, PR China; ^3^​ School of Veterinary Medicine and Science, University of Nottingham – Sutton Bonington Campus, Sutton Bonington, UK

## Abstract

The PA-X protein, arising from ribosomal frameshift during PA translation, was recently discovered in influenza A virus (IAV). The C-terminal domain ‘X’ of PA-X proteins in IAVs can be classified as full-length (61 aa) or truncated (41 aa). In the main, avian influenza viruses express full-length PA-X proteins, whilst 2009 pandemic H1N1 (pH1N1) influenza viruses harbour truncated PA proteins. The truncated form lacks aa 232–252 of the full-length PA-X protein. The significance of PA-X length in virus function remains unclear. To address this issue, we constructed a set of contemporary influenza viruses (pH1N1, avian H5N1 and H9N2) with full and truncated PA-X by reverse genetics to compare their replication and host pathogenicity. All full-length PA-X viruses in human A549 cells conferred 10- to 100-fold increase in viral replication and 5–8 % increase in apoptosis relative to corresponding truncated PA-X viruses. Full-length PA-X viruses were more virulent and caused more severe inflammatory responses in mice. Furthermore, aa 233–252 at the C terminus of PA-X strongly suppressed co-transfected gene expression by ∼50 %, suggesting that these terminal 20 aa could play a role in enhancing viral replication and contribute to virulence.

## Introduction

Influenza A virus (IAV) causes considerable economic loss to the global livestock industry and poses a significant threat to public health. IAV is an enveloped negative-strand RNA virus with eight viral RNA segments encoding PB2, PB1, PA, HA, NP, NA, M1, M2, NS1 and NS2. In recent years, several additional proteins were discovered, such as PB1-F2, N40, PA-N155 and PA-N182 (Chen *et al.*, 2001; Muramoto *et al.*, 2013; Vasin *et al.*, 2014; Wise *et al.*, 2009). Previous studies showed that NS1 and PB1-F2 proteins of different influenza viruses have varying lengths that differentially affect viral properties such as pathogenicity (Hai *et al.*, 2010; Wang *et al.*, 2005; Zell *et al.*, 2007). The virulence of H1N1 and H3N2 viruses, with truncated NS1 proteins at the C terminus, in mice and pigs was attenuated, unlike full-length NS1 virus infections (Kochs *et al.*, 2009; Solórzano *et al.*, 2005). H9N2 viruses with different forms of NS1 showed varying replication rates and transmission ability, and induced different levels of inflammatory cytokines in chickens (Kong *et al.*, 2015). Extension of PB1-F2 from a truncated 11 aa to full-length in pandemic 2009 H1N1 virus (pH1N1) moderately enhanced viral replication and cytokine response in pigs (Pena *et al.*, 2012).

Recently, it was demonstrated that segment 3 of IAV encodes not only the PA protein, but also an additional novel protein, PA-X, which arises from ribosomal frameshift in a — +1 ORF (X ORF) extension of a growing PA polypeptide (Jagger *et al.*, 2012; Yewdell & Ince, 2012). PA-X is a hybrid protein that comprises the N-terminal domain of PA (191 aa) and the C-terminal domain X. PA-X in the 1918 pandemic H1N1 virus reduces viral pathogenicity and modulates host responses in mice; PA-X also plays an important role in host gene expression shutoff characterized by inhibition of host protein synthesis (Desmet *et al.*, 2013; Jagger *et al.*, 2012). Comprehensive genetic analysis showed that the X ORFs of diverse IAVs can be divided into two groups according to protein length (Shi *et al.*, 2012). Avian, equine and human seasonal H3N2 and H1N1 influenza viruses express a full-length PA-X protein encoded by 252 aa with 61 aa of the X ORF. In contrast, some IAVs, including human pH1N1, canine and certain swine influenza viruses possess a TGG (Trp) to TAG (stop codon) mutation at codon 42 in the X ORF. This mutation confers a truncated PA-X protein encoded by 232 aa with 41 aa of the X domain. The effects of PA-X length on the biological characteristics of IAVs remain unclear.

In the present study, we used pH1N1, a highly pathogenic avian influenza virus of the H5N1 subtype, and a low pathogenicity avian H9N2 influenza virus as prevalent exemplars of truncated human and full-length avian PA-X viruses to examine the contribution of the C-terminal end, from aa 233 to 252, of PA-X in these influenza viruses to viral replication and pathogenicity in mammalian cells and mice. We found that viruses with full-length PA-X replicated more efficiently and were more pathological in A549 cells and mice than the corresponding viruses with truncated PA-X.

## Results

### Generation of pH1N1, H5N1 and H9N2 viruses encoding full-length PA-X and truncated PA-X

**Fig. 1.**
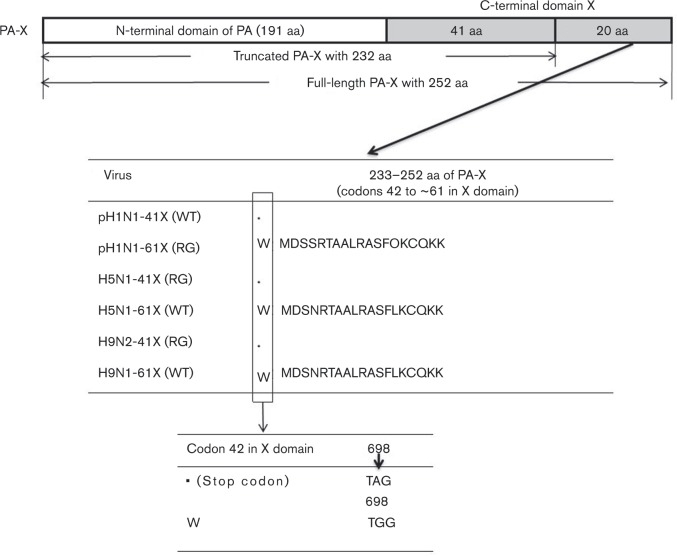
Sequences of the C-terminal regions of PA-X proteins of the virus strains used in the study. H5N1 and H9N2 viruses expressed a full-length PA-X protein. In contrast, pH1N1 virus had G698A, which is a TGG (W)-to-TAG (stop codon) mutation at codon 42 in the X ORF, producing a truncated PA-X protein with 41 aa in the X domain. Amino acids 233–252 in the C terminus of all PA-X mutant viruses used in the study are shown. pH1N1-61X (RG) expressing full-length PA-X was generated by introducing a A698G mutation which converted the stop codon to W (tryptophan). H5N1-41X (RG) and H9N2-41X (RG) expressing truncated PA-X were generated by introducing a G698A mutation which was a W-to-stop codon mutation at codon 42 in the PA-X ORF of H5N1-61X (WT) and H9N2-61X (WT) viruses. A698G and G698A mutations did not affect the PA ORF.

Sets of influenza viruses based on human truncated PA-X A/Beijing/16/2009 (BJ/09, pH1N1), and avian full-length PA-X A/tree sparrow/Jiangsu/1/2008 (JS08, H5N1) and A/chicken/Hebei/LC/2008 (HB/08, H9N2), viruses were generated using reverse genetics. Avian H5N1 and H9N2 influenza viruses each express a full-length 252 aa PA-X protein with 61 aa in the X domain, whilst pH1N1 virus has a G698A stop codon mutation at codon 42 in the X ORF (Fig. 1). This stop codon produces a truncated 232 aa PA-X protein with 41 aa in the X domain. To assess the function of the C terminus (aa 233–252) of PA-X, pH1N1-61X (RG) was generated by the introduction of an A698G mutation in the PA-X ORF of pH1N1-41X (WT). Truncated PA-X viruses H5N1-41X (RG) and H9N2-41X (RG) were generated by the introduction of a G698A mutation in the PA-X ORF of H5N1-61X (WT) and H9N2-61X (WT) viruses. A698G and G698A mutations did not affect the PA ORF (Fig. 1).

### Amino acids 233–252 at the C terminus of full-length PA-X play a role in viral replication and apoptosis in human lung cells

**Fig. 2.**
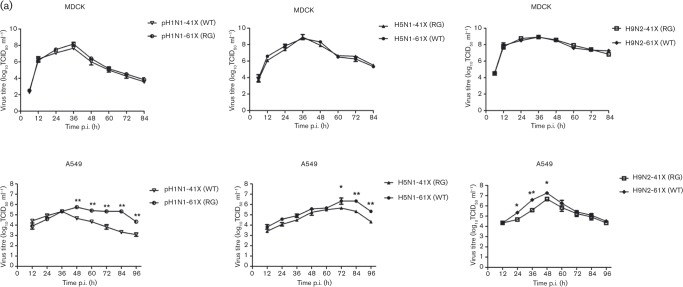

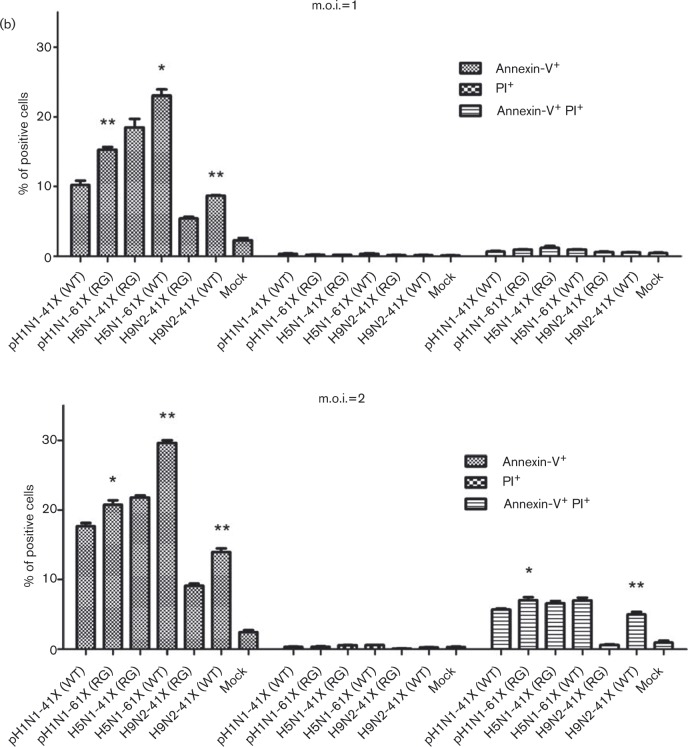
Viruses with full-length PA-X replicated more efficiently and induced more apoptosis in A549 cells than the corresponding viruses with truncated PA-X. (a) Viral growth curves of pH1N1, H5N1 and H9N2 mutant viruses in MDCK cells and A549 cells over 84 or 96 h. Data represent the mean ± sd of three independent experiments. (b) Relative induction of apoptosis/cell death as determined by the detection of annexin-V^+^, PI^+^ and annexin-V^+^ PI^+^ in A549 cells infected with the indicated panel of viruses at m.o.i. 1.0 and 2.0 for 12 h. Mock, uninfected control cells. Data represent the mean ± sd of three independent experiments performed in triplicates. Significant differences between pH1N1-61X (RG) and pH1N1-41X (WT), H5N1-41X (RG) and H5N1-61X (WT), and H9N2-41X (RG) and H9N2-61X (WT): *P < 0.05; **P < 0.01.

The six viruses were used to infect Madin–Darby canine kidney (MDCK) and human lung (A549) cells at m.o.i. 0.01, and the supernatants were collected and titrated at 12, 24, 36, 48, 60, 72, 84 and 96 h post-infection (p.i.). There was no significant difference in viral output from MDCK cells between the two forms of pH1N1, H5N1 and H9N2 viruses (Fig. 2a). In contrast, with A549 cells, the levels of pH1N1-61X (RG) virus with full-length PA-X were 10^1^–10^2^ TCID_50_ higher than those with truncated PA-X [pH1N1-41X (WT)] from 48 h p.i. (*P* < 0.01) (Fig. 2a). H5N1-61X (WT) with full-length PA-X showed 10^0.67^–10^1^ TCID_50_ higher virus production than H5N1-41X (RG) with truncated PA-X at 72, 84 and 96 h p.i. (*P* < 0.05). Full-length PA-X virus of H9N2-61X (WT) produced more progeny virus (∼10^1^ TCID_50_) than H9N2-41X (RG) at 24, 36 and 48 h p.i. (*P* < 0.05). Collective data from A549 cells indicated that pH1N1, H5N1 and H9N2 viruses with full-length PA-X replicated more efficiently than the corresponding viruses with truncated PA-X.

Several viral proteins (NA, M1, NS1 and PB1-F2) have been shown to promote apoptosis, associated with cellular and organ damage (Chanturiya *et al.*, 2004; Chen *et al.*, 2001; Morris *et al.*, 1999; Zhirnov *et al.*, 2002a, b). To evaluate the effect of the aa 233–252 portion at the C terminus of PA-X on apoptosis and necrosis, A549 cells were infected with the panel of recombinant viruses at m.o.i. 1.0 and 2.0 for 12 h. Detection of apoptosis by flow cytometry for only annexin-V^+^ cells found that pH1N1-61X (RG) virus caused significantly more apoptosis (15.31 % at m.o.i. 1.0 and 20.77 % at m.o.i. 2.0) than the corresponding pH1N1-41X (WT) virus (10.24 % at m.o.i. 1.0 and 17.70 % at m.o.i. 2.0) (*P* < 0.01 or *P* < 0.05) (Figs 2b and S1, available in the online Supplementary Material). Similarly, H5N1-61X (WT) virus produced more apoptotic cells (23.03 % at m.o.i. 1.0 and 29.63 % at m.o.i. 2.0) than the corresponding H5N1-41X (RG) virus (18.47 % at m.o.i. 1.0 and 21.80 % at m.o.i. 2.0). H9N2-41X (RG) virus produced fewer apoptotic cells (5.44 % at m.o.i. 1.0 and 9.09 % at m.o.i. 2.0) than the corresponding H9N2-61X (WT) virus (8.68 % at m.o.i. 1.0 and 13.93 % at m.o.i. 2.0) (*P* < 0.01 or *P* < 0.05). Detection of both apoptosis and necrosis by flow cytometry for annexin-V^+^ and propidium iodide (PI)^+^ cells at m.o.i. 2.0 showed that pH1N1-61X (RG) virus induced more cell death (7.07 %) than pH1N1-41X (WT) virus (5.70 %), and H9N2-61X (WT) virus caused more cell death (4.98 %) than H9N2-41X (RG) virus (0.601 %) (*P* < 0.01 or *P* < 0.05). In summary, viruses with full-length PA-X induced greater apoptosis, and to some extent more necrosis, in A549 cells relative to truncated PA-X for pH1N1, H5N1 and H9N2 viruses.

### Viruses with full-length PA-X are more virulent than those with truncated PA-X in mice

**Fig. 3.**
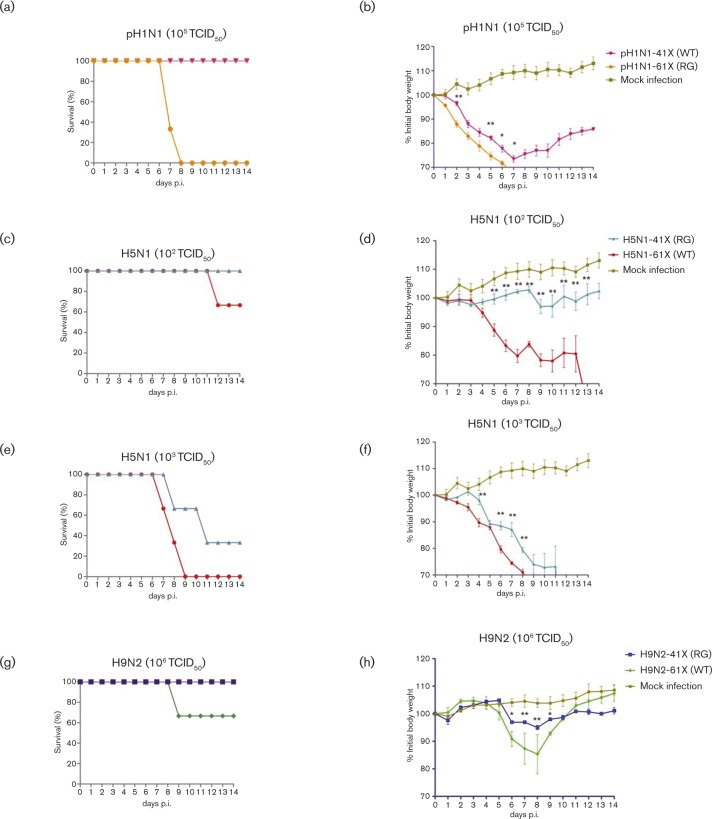
Full-length PA-X viruses were more virulent than the corresponding truncated PA-X viruses in mice. Data show the survival (%) of mice infected with 10^5^ TCID_50_ pH1N1 (a), 10^2^ or 10^3^ TCID_50_ H5N1 (c, e) and 10^6^ TCID_50_ H9N2 PA-X mutant viruses (g). The body weight of mice inoculated with pH1N1 (b), H5N1 (d, f) and H9N2 (h) PA-X mutant viruses is presented as percentage of the weight on the day of inoculation (day 0). Any mouse that lost >30 % of its body weight was euthanized. Data represent the mean ± sd of the body weight of three mice per group (*n* = 3).

**Fig. 4.**
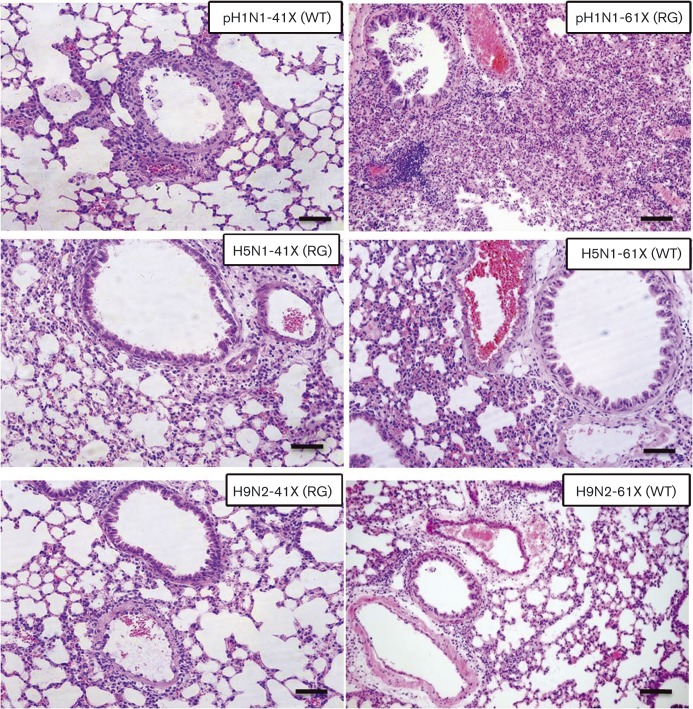
Viruses with full-length PA-X caused more severe lung lesions than the corresponding viruses with truncated PA-X. Histopathological changes in mice infected with PA-X mutant viruses. A portion of lung from mice infected with 10^5^ TCID_50_ pH1N1, 10^3^ TCID_50_ H5N1 or 10^6^ TCID_50_ H9N2 mutant viruses at 5 days p.i. was fixed in 10 % phosphate-buffered formalin and processed for paraffin embedding. Each 5 μm section was stained with haematoxylin and eosin, and examined for histopathological changes. Bars, 100 μm.

**Fig. 5.**
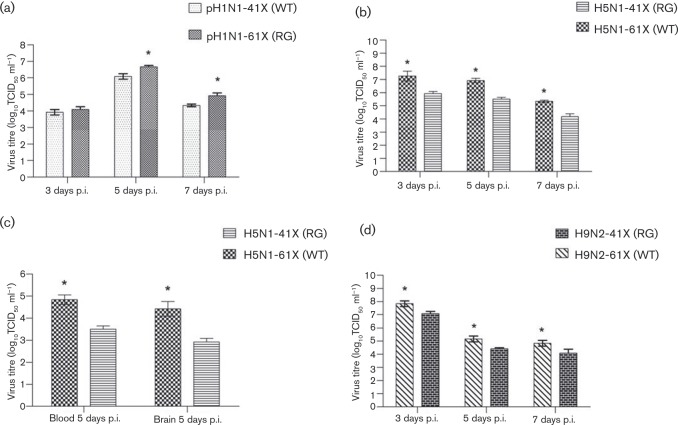
Viruses with full-length PA-X showed higher replication levels in mice than the corresponding viruses with truncated PA-X. The mean ± sd viral load was calculated by log_10_TCID_50_ determination in MDCK cells. Three mice per group were infected with 10^5^ TCID_50_ pH1N1 (a), 10^3^ TCID_50_ H5N1 (b, c) or 10^6^ TCID_50_ H9N2 mutant viruses (d). Data represent the mean ± sd of the viral titres of three mice (*n* = 3). Significant difference between pH1N1-61X (RG) and pH1N1-41X (WT), H5N1-61X (WT) and H5N1-41X (RG), and H9N2-61X (WT) and H9N2-41X (RG): *P < 0.05.

To assess the pathogenicity of PA-X variants, 6-week-old BALB/c mice (15 mice per group) were intranasally inoculated with each of the six viruses. Clinical signs, mortality and weight loss were monitored over 14 days. Three infected mice were humanely killed at 3, 5 and 7 days p.i., and lungs were collected for virus titration. For pH1N1 mutants, survival curves at 10^5^ TCID_50_ clearly showed that pH1N1-61X (RG) had a higher pathogenicity index than pH1N1-41X (WT) (Fig. 3a). pH1N1-61X (RG) conferred 100 % mortality by 8 days p.i.; pH1N1-41X (WT) caused no death. The loss of body weight due to pH1N1-61X (RG) was greater than that attributed to pH1N1-41X (WT) (Fig. 3b). The pathogenicity of these viruses was further assessed by histopathology of lung tissues collected at 5 days p.i. As shown in Fig. 4, pH1N1-61X (RG) caused more severe lesions than pH1N1-41X (WT), exhibiting extensive alveolar damage and cellular infiltration. pH1N1-61X (RG) also showed higher viral replication in the lung than pH1N1-41X (WT) at 5 and 7 days p.i. (*P* < 0.05) (Fig. 5a).

With H5N1 viruses, H5N1-41X (RG), at 10^2.75^ TCID_50_ mean lethal dose (MLD_50_), was more attenuated in mice than the corresponding H5N1-61X (WT) with MLD_50_ at 10^2.25^ TCID_50_. Body weight loss of H5N1-41X (RG)-infected mice was less than that of H5N1-61X (WT)-infected mice after inoculation at 10^2^ or 10^3^ TCID_50_ (Fig. 3d, f). Survival curves showed that H5N1-61X (WT) was more pathogenic than H5N1-41X (RG) (Fig. 3c, e). H5N1-61X infection resulted in 100 % mortality by 9 days p.i.; H5N1-41X (RG) caused 66.6 % mortality after inoculation at 10^3^ TCID_50_ (Fig. 3c, e). H5N1-61X (WT) also showed greater pathogenicity than H5N1-41X (RG), with 33.3 % mortality by 12 days p.i.; H5N1-41X (RG) caused no death after inoculation at 10^2^ TCID_50_. Mice infected with H5N1-61X (WT) showed more severe lung lesions than those infected with H5N1-41X (RG), characterized by interstitial oedema, thickening of the alveolar walls and cellular infiltration (Fig. 4). Viral titres of H5N1-41X (RG) in lungs were significantly lower than those of H5N1-61X (WT) at 3, 5 and 7 days p.i. (*P* < 0.05) (Fig. 5b). Previous studies showed that infections with pH1N1 and H9N2 viruses were restricted to the respiratory system, whilst H5N1 viruses could result in systemic infection and neurovirulence in mice (Sun *et al.*, 2011; Wang *et al.*, 2012; Zhang *et al.*, 2012). H5N1-61X (WT) indeed showed higher replication levels in the brain and blood than the corresponding H5N1-41X (RG) at 5 days p.i. (*P* < 0.05) (Fig. 5c).

As evident in the survival curves of mice infected with H9N2 viruses at 10^6^ TCID_50_, H9N2-41X (RG) infection caused no death, but H9N2-61X (WT) infection resulted in 33.3 % mortality (Fig. 3g). The maximum weight loss from H9N2-61X (WT) and H9N2-41X (RG) infection was 12 and 5 %, respectively (Fig. 3h). Lungs infected with H9N2-41X (RG) virus appeared almost normal, but mild pathological changes were observed in the H9N2-61X (WT) group (Fig. 4). H9N2-61X (WT)-infected lungs showed some inflammatory cells in blood vessels and epithelial erosion of bronchial lining. The viral titres of lungs infected with H9N2-41X (RG) were lower than those infected with H9N2-61X (WT) at 3, 5 and 7 days p.i. (*P* < 0.05) (Fig. 5d), consistent with the observed pathology. Collectively, these results indicated that pH1N1, H5N1 and H9N2 viruses with full-length PA-X were more pathogenic than the corresponding subtypes with truncated PA-X.

### Viruses with full-length PA-X cause more severe inflammatory responses in mice than those with truncated PA-X

Severe influenza virus infection in humans and animal models is associated with abnormally elevated pulmonary pro-inflammatory cytokine and chemokine expression (Bermejo-Martin *et al.*, 2010; Hagau *et al.*, 2010; Lam *et al.*, 2010; Perrone *et al.*, 2008). To assess the effect of the aa 233–252 portion in the C terminus of PA-X on host inflammatory response, protein levels of seven cytokines and chemokines in the lungs of infected mice were determined at 3 and 5 days p.i.

**Fig. 6.**
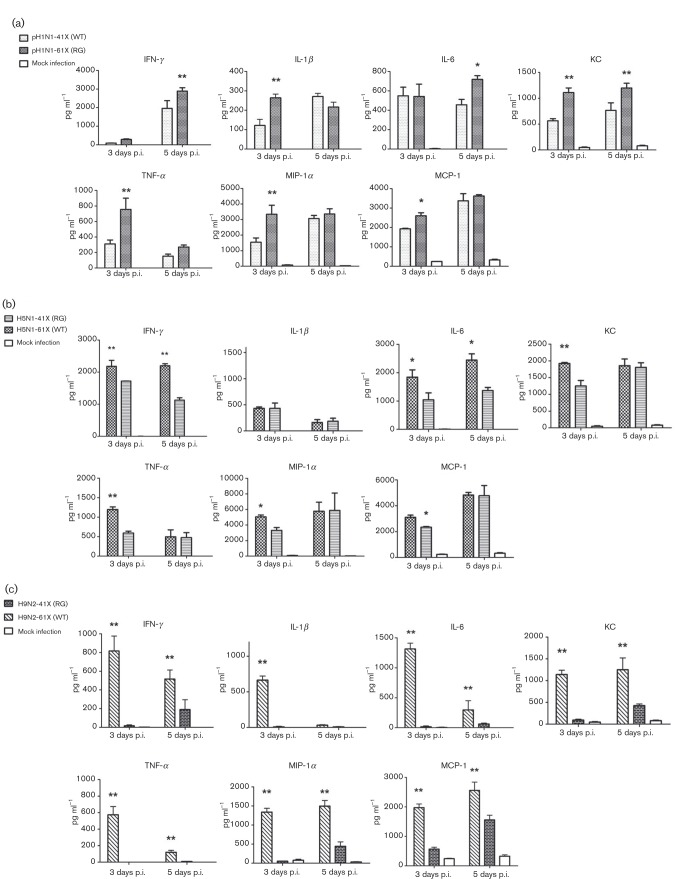
The aa 233–252 portion at the C terminus of full-length PA-X promoted lung inflammation in mice. Detection of cytokine and chemokine proteins in lungs of mice infected with pH1N1 (a), H5N1 (b) and H9N2 (c) PA-X mutant viruses. Data represent the mean ± sd cytokine/chemokine levels (*n* = 3). *Significant differences between viruses with full-length PA-X and truncated PA-X: *P < 0.05; **P < 0.01.

Mice infected with pH1N1-61X (RG) virus exhibited higher titres of IFN-γ, IL-1β, IL-6, mouse IL-8 (KC), TNF-α, macrophage inflammatory protein (MIP)-1α and monocyte chemotactic protein (MCP)-1 than those with pH1N1-41X (WT) at 3 or 5 days p.i. (*P* < 0.05 or *P* < 0.01) (Fig. 6a). Mice infected with H5N1-61X (WT) virus showed higher levels of IFN-γ, IL-6, KC, TNF-α, MIP-1α and MCP-1 than H5N1-41X (RG) virus at 3 or 5 days p.i. (*P* < 0.05 or *P* < 0.01) with the exception of IL-1β (Fig. 6b). With H9N2 viruses, all the chemokine and cytokine levels of H9N2-61X (WT) virus were higher than those of H9N2-41X (RG) virus at 3 or 5 days p.i. (*P* < 0.05 or *P* < 0.01) (Fig. 6c).

These results showed that infection with pH1N1, H5N1 and H9N2 viruses housing full-length PA-X elicited a more severe inflammatory response than infection with the corresponding truncated PA-X viruses.

### Amino acids 233–252 at the C terminus of PA-X strongly suppress co-transfected gene expression

**Fig. 7.**
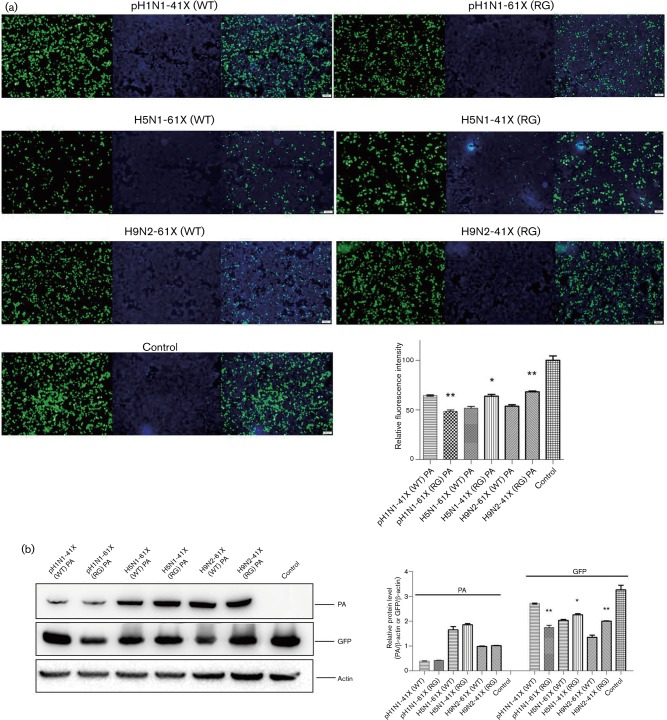
PA plasmids with full-length PA-X were more effective in suppressing co-transfected gene expression than those with truncated PA-X. (a) 293T cells were co-transfected with EGFP expression plasmid and PA plasmids with full-length or truncated PA-X from pH1N1, H5N1 and H9N2 viruses. Fluorescence images indicative of EGFP expression at 24 h after transfection were captured under identical exposure conditions. Left panel, GFP fluorescence (green); middle panel, DAPI staining (blue); right panel, merged fluorescence. Control was 293T cells co-transfected with EGFP expression plasmid and empty pcDNA3.1 vector. Fluorescence intensities were determined with Image-Pro Plus (Media Cybernetics). Relative fluorescence intensities of each group relative to control are shown as histograms. Data represent the mean ± sd of three independent experiments. (b) PA protein and GFP were determined by Western blotting using PA and GFP antibodies. β-Actin antibody was used as loading control. Protein bands were quantified by densitometry. Relative protein levels of PA and GFP normalized to β-actin are shown as histograms. Data represent the mean ± sd of three independent experiments. Significant differences between viruses with full-length PA-X and truncated PA-X: *P < 0.05; **P < 0.01.

Influenza virus infection can result in host gene expression shutoff, whereby the virus controls cellular transcription/translation machinery for the preferential production of viral proteins (Katze *et al.*, 1986a, b). Inhibition of host protein synthesis can hinder the cellular antiviral response, contributing to efficient virus production and spread. The PA-X protein is known to play a major role in the suppression of host protein synthesis (Desmet *et al.*, 2013; Jagger *et al.*, 2012). In order to determine the role of the aa 233–252 portion at the C terminus of PA-X in protein synthesis inhibition, the suppression activity of PA plasmids with full-length PA-X was compared with that of truncated PA-X of pH1N1, H5N1 and H9N2 viruses by co-transfection with pEGFP expression plasmid in human embryonic kidney (293T) cells over 24 h. PA and GFP expression was determined by fluorescence intensity and Western blotting (Fig. 7a, b). There was significantly less EGFP expression in the presence of PA plasmids with full-length PA-X than in the presence of the corresponding PA plasmids with truncated PA-X of pH1N1, H5N1 and H9N2 viruses (*P* < 0.05 or *P* < 0.01). The levels of PA expression were similar between PA plasmids with full-length and truncated PA-X in the three virus strains. The differences of EGFP expression did not correlate with the levels of PA expression.

**Fig. 8.**
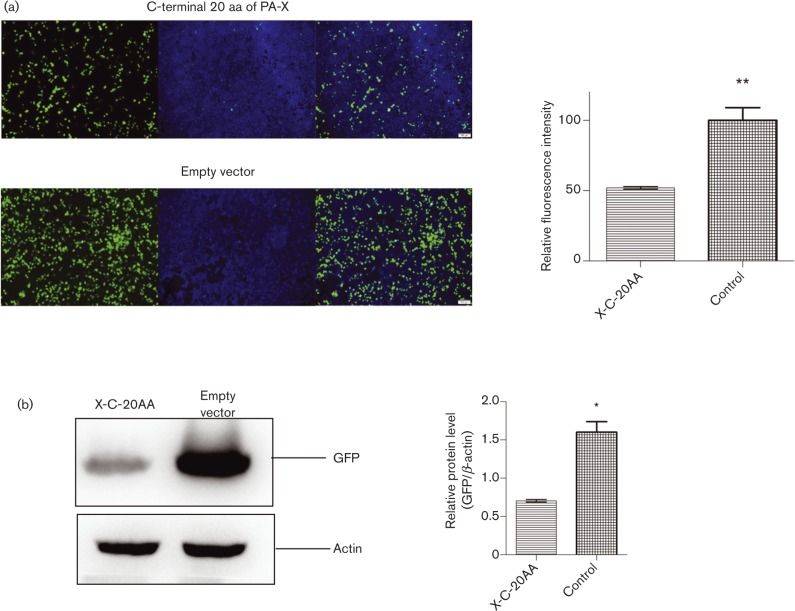
The aa 233–252 portion of PA-X alone strongly suppressed co-transfected gene expression. (a) 293T cells were co-transfected with EGFP expression plasmid and the C-terminal 20 aa of the full-length PA-X plasmids (X-C-20AA; sequence WMDSNRTAALRASFLKCQKK). Fluorescence images indicative of EGFP expression at 24 h after transfection were captured under identical exposure conditions. Left panel, GFP fluorescence (green); middle panel, DAPI staining (blue); right panel, merged fluorescence. The fluorescence intensities were determined with Image-Pro Plus (Media Cybernetics). Relative fluorescence intensities of each group relative to control are shown as histograms. Data represent the mean ± sd of three independent experiments. (b) GFP expression was determined by Western blotting using GFP antibody. β-Actin antibody was used as loading control. Relative protein levels of GFP relative to β-actin are shown as histograms. Data represent the mean ± sd of three independent experiments. Significant differences between viruses with full-length PA-X and truncated PA-X: *P < 0.05; **P < 0.01.

As PA plasmids with full-length PA-X plasmids rather than the corresponding truncated PA-X plasmids were more effective in suppressing co-transfected EGFP gene expression, it was possible that the aa 233–252 portion in the C terminus of PA-X was responsible for the suppression of EGFP expression. To address this, we constructed a plasmid that expressed only the C-terminal 20 aa portion of full-length PA-X. Co-transfection of this construct with pEGFP showed a clear reduction in EGFP production of ∼50 % (Fig. 8a, b). Therefore, the C-terminal 20 aa of full-length PA-X were important in co-transfected gene suppression.

## Discussion

In the present study, influenza viruses with full-length PA-X showed higher replication output and pathogenicity than those with truncated PA-X in human lung cells and mice for pH1N1, H5N1 and H9N2 viruses. Moreover, we found that aa 233–252 at the C terminus of PA-X could strongly suppress co-transfected gene expression, which might be responsible for enhanced viral replication and virulence.

Full-length and truncated forms of PA-X in pH1N1, H5N1 and H9N2 viruses showed no significant difference in virus replication in MDCK cells. Jagger *et al.* (2012) reported similar findings in MDCK cells, which showed no difference in single- or multi-cycle growth kinetics of the 1918 H1N1 PA-X-deficient and WT PA-X viruses (Jagger *et al.*, 2012). In contrast, we found that pH1N1, H5N1 and H9N2 viruses with full-length PA-X showed higher replication levels in A549 cells than those with truncated PA-X. Challenge experiments showed that viruses with full-length PA-X exhibited greater pathogenicity (mortality and lung pathology) than those with truncated PA-X for pH1N1, H5N1 and H9N2 viruses. In addition, viruses with full-length PA-X showed higher replication levels in murine lungs. These results indicate that increased replication and pathogenicity in mice are features of viruses with full-length PA-X.

Severe influenza virus infection can go through a direct pathogenic pathway by inducing apoptosis, leading to cell death and loss of critical function, or more likely through an indirect pathogenic pathway of inducing excessive cytokine/chemokine production from the infected cells (Korteweg & Gu, 2008). Jagger *et al.* (2012) found that loss of PA-X expression led to changes in the kinetics of the global host gene response, which notably showed increases in inflammatory, apoptotic and T-lymphocyte signalling pathways during infection with the 1918 pandemic virus. In contrast, we showed that the C-terminal 20 aa of the full-length PA-X promoted inflammatory response in mice and apoptosis in A549 cells with pH1N1, H5N1 and H9N2 virus infection. Cytokine and chemokine responses, including those of IL-1β, IFN-γ, TNF-α, MIP-1α, IL-6, MIP-2, MCP-1, KC and IL-1α, are found to associate with the recruitment of macrophages and neutrophils to infected lungs, which result in acute lung inflammation (Perrone *et al.*, 2008). In the present study, virtually all the chemokine and cytokine levels of full-length PA-X viruses examined were significantly higher than those of the corresponding truncated PA-X viruses. Therefore, full-length PA-X viruses elicited more severe pro-inflammation and pathogenicity than the corresponding truncated PA-X viruses.

Apoptosis plays an important role in the pathogenesis of many infectious diseases, including those caused by viruses (Ludwig *et al.*, 1999; Young *et al.*, 1997). Influenza viruses have been reported to induce apoptosis in numerous cell types, both *in vivo* and *in vitro* (Mori *et al.*, 1995; Yang *et al.*, 2011). Several viral proteins have been shown to promote or inhibit apoptosis, such as NS1 and PB1-F2 (Chanturiya *et al.*, 2004; Chen *et al.*, 2001; Zhirnov *et al.*, 2002a). The C-terminal portion of the PB1-F2 ORF contains a mitochondrial targeting sequence (Chen *et al.*, 2001). Expression of full-length PB1-F2 has been associated with mitochondrial targeting and apoptosis, and it has been suggested that mitochondrial disruption with subsequent cell death could contribute to virulence (Chen *et al.*, 2001; Gibbs *et al.*, 2003; Zamarin *et al.*, 2005). It has also been demonstrated that NS1 proteins of different subtypes of influenza viruses promote apoptosis-inducing ability (Brydon *et al.*, 2005; Lam *et al.*, 2011). Here, we found that viruses with full-length PA-X induced increases in apoptosis in A549 cells for pH1N1, H5N1 and H9N2 viruses. The efficient replication of viruses with full-length PA-X in lung cells, and the induction of inflammation and apoptosis, could cause severe lung damage and reduce lung function.

Influenza virus infection can induce host cell shutoff, which is characterized by a rapid decline of host protein synthesis (Katze *et al.*, 1986a, b). Inhibition of cellular gene expression limits the induction of an antiviral response and diverts ribosomes towards translation of viral mRNAs. NS1 can inhibit type I IFN synthesis, block nuclear export of mRNA and inhibit mRNA splicing (Fortes *et al.*, 1994; García-Sastre *et al.*, 1998; Talon *et al.*, 2000). PA has been shown to suppress host protein synthesis. Desmet *et al.* (2013) showed that the N-terminal domain of PA, which includes the endonuclease active site, is sufficient to suppress protein expression and the suppressive effect of the N-terminal domain of PA was mainly due to PA-X. Their further results showed that the C-terminal X domain of PA-X also played a major role in the suppression of protein expression. In our study, PA plasmids with full-length PA-X were more effective in suppressing co-transfected gene expression than those with truncated PA-X. Moreover, co-transfection of a plasmid expressing only the C-terminal 20 aa of full-length PA-X with pEGFP showed reduced EGFP expression. Our data suggest that the aa 233–252 portion at the C terminus of PA-X is involved in the suppression of host protein synthesis, and contributes to enhanced viral replication and pathogenicity in full-length PA-X viruses.

Variations in PA-X protein length in IAVs have been observed in nature. Avian influenza viruses are believed to be progenitors of all IAVs and full-length PA-X is conserved in avian influenza viruses (Shi *et al.*, 2012). Adaptation in the form of truncation of PA-X could have occurred when avian influenza viruses were introduced into certain new hosts. For example, PA-X truncation occurred after avian H3N2 viruses were introduced into canines (Shi *et al.*, 2012). A group of older 1930–2006 classical swine H1N1 viruses express full-length PA-X, whilst a cluster of classical swine H1N1 viruses sampled between 1985 and 2009 express truncated PA-X after the adaptation in pigs. There may be some species specificity to the evolution of a truncated PA-X protein. Further experiments are needed to explore the relationship between PA-X truncation and host specificity.

## Methods

### Viruses and cells

A/Beijing/16/2009 (BJ/09, pH1N1), A/tree sparrow/Jiangsu/1/2008(JS08, H5N1) and A/chicken/Hebei/LC/2008 (HB/08, H9N2) viruses were as described previously (Liu *et al.*, 2010; Sun *et al.*, 2011). Human 293T, MDCK and human pulmonary adenocarcinoma cells (A549) were maintained in Dulbecco's modified Eagle's medium (Life Technologies) supplemented with 10 % FBS (Life Technologies), 100 U penicillin ml^− 1^ and 100 g streptomycin ml^− 1^.

Experiments with H5N1 influenza virus were performed in a Biosafety Level 3 containment laboratory approved by the Ministry of Agriculture of the People's Republic of China.

### Generation of recombinant viruses by reverse genetics

All eight gene segments were amplified by reverse transcription (RT)-PCR from BJ/09, JS/08 and HB/08 viruses, and cloned into the dual-promoter plasmid pHW2000. The mutations were introduced into the PA gene using a Site-directed QuikChange Mutagenesis kit (Agilent) according to the manufacturer's instructions. PCR primer sequences are available upon request. pH1N1 with full-length PA-X (61 aa), pH1N1-61X (RG), had a stop (UAG)-to-tryptophan (UGG) codon substitution at position 42 in the X ORF (stop42W) without changing the PA ORF (Fig. 1b). H5N1 and H9N2 viruses with truncated PA-X (41 aa), H5N1-41X (RG) and H9N2-41X (RG), had a tryptophan (UGG)-to-stop (UAG) codon substitution at position 42 in the X ORF (W42stop) without altering the PA ORF. Rescued viruses were detected using haemagglutination assays. The viruses were purified by sucrose density gradient centrifugation. Viral RNA was extracted and analysed by RT-PCR, and each viral segment was sequenced to confirm sequence identity.

### Viral titration and replication kinetics

TCID_50_ was determined in MDCK cells using 10-fold serially diluted virus inoculated at 37 °C and cultured for 72 h. The TCID_50_ values were calculated using the method first described by Reed & Muench (1938).

MDCK and A549 cells were infected with virus at m.o.i. 0.01. Supernatants of the infected MDCK cells were harvested at 6, 12, 24, 36, 48, 60, 72 and 84 h p.i. Supernatants of the infected A549 cells were harvested at 12, 24, 36, 48, 60, 72, 84 and 96 h p.i. Viral titres were determined in MDCK cells from the TCID_50_. Three independent experiments were performed.

### Mouse infection

Fifteen mice (6-week-old female BALB/c; Vital River Laboratory) per group were anaesthetized with Zoletil (tiletamine–zolazepam; Virbac; 20 μg g^− 1^) and inoculated intranasally with 50 μl 10^5^ TCID_50_ pH1N1, 10^2^ or 10^3^ TCID_50_ H5N1 or 10^6^ TCID_50_ H9N2, diluted in PBS. All mice were monitored daily for 14 days; mice that lost 30 % of their original body weight were humanely euthanized. Three mice were euthanized 3, 5 and 7 days p.i. for determination of lung virus titres, histology and cytokine levels. Lungs, brains and blood were collected and homogenized in cold PBS. Virus titres were determined by TCID_50_. MLD_50_ values were determined by intranasally inoculating groups of three mice with 10-fold dilutions of virus and were calculated by the method of Reed & Muench (1938). Animal research was approved by the Beijing Association for Science and Technology, and complied with the Beijing laboratory animal welfare and ethical guidelines of the Beijing Administration Committee of Laboratory Animals.

### Histopathology

At 5 days p.i., a portion of lung from each euthanized mouse was fixed in 10 % phosphate-buffered formalin and processed for paraffin embedding. Each 5 μm section was stained with haematoxylin and eosin and examined for histopathological changes. Images were captured with a Zeiss Axioplan 2IE epifluorescence microscope.

### Quantification of cytokine and chemokine protein levels in mouse lungs

Levels of cytokines and chemokines, including IL-1β, IL-6, KC, MCP-1, MIP-1α, TNF-α, and IFN-γ, in the lung were determined using a cytometric bead array method (BD Cytometric Bead Array Mouse Inflammation kit; BD Bioscience). Briefly, 50 μl mouse inflammation capture bead suspension and 50 μl detection reagent were added to an equal amount of sample and incubated in the dark for 2 h at room temperature. Samples were later washed with 1 ml wash buffer and then centrifuged at 200 ***g*** at room temperature for 5 min. Supernatants were discarded and 300 μl wash buffer was added. Samples were analysed on a BD FACSArray bioanalyser (BD Bioscience). Data were analysed using BD Cytometric Bead Array software (BD Bioscience). Chemokine and cytokine levels were recorded as pg ml^− 1^ in the homogenate.

### Cell death assays

Virus infection assays were conducted in six-well plates. Cells were seeded at a density of 1 × 10^6^ cells per well overnight in infection media (cell growth media with 1 % BSA was used in place of FCS). Cells were then infected with virus at m.o.i. 1.0 for 12 h. Cells from the supernatant and monolayers were then harvested, washed and stained with allophycocyanin-labelled annexin and PI (Becton Dickinson) for 20 min. After a final wash step, cells were resuspended in 100 μl FACS wash buffer (PBS containing 3 % BSA and 0.01 % sodium azide), and analysed using a FACSCalibur (BD Biosciences) and Flow Jo software (version 7.6.1). Cell death (apoptosis and necrosis) was defined as annexin-V^+^ and PI^+^, whilst apoptotic cells were annexin-V^+^ only. Viable cells were considered neither annexin-V^+^ nor PI^+^.

### Western blotting

Total cell protein lysates were extracted from transfected 293T cells and infected MDCK cells with CA630 lysis buffer (150 mM NaCl, 1 % CA630 detergent, 50 mM Tris base, pH 8.0). Cellular proteins were separated by 12 % SDS-PAGE and transferred to a PVDF membrane (Amersham Biosciences). Each PVDF membrane was blocked with 0.1 % Tween 20 and 5 % non-fat dry milk in Tris-buffered saline and subsequently incubated with a primary antibody. Primary antibodies were specific to IAV PA (1 : 3000; GeneTex) and GFP (1 : 1000; Abcam). Secondary antibodies included HRP-conjugated anti-mouse antibody or HRP-conjugated anti-rabbit antibody (1 : 10 000; Jackson ImmunoResearch), as appropriate. HRP presence was detected using a Western Lightning Chemiluminescence kit (Amersham Pharmacia) in accordance with the manufacturer's protocol. Protein bands were quantified by densitometry using Image Studio Digits (LI-COR Biosciences).

### Statistical analysis

The statistical analyses were performed using Prism version 5.00 (GraphPad). Comparison between two forms of treatment involved the two-tailed Student's *t*-test; multiple comparisons were carried out using two-way ANOVA considering time and virus as factors. Differences were considered statistically significant at *P* < 0.05.

## Supplementary Data

180 Supplementary Data
