# Supplementary material for: Twenty amino acids at the C-terminus of PA-X are associated with increased influenza A virus replication and pathogenicity
Source: J Gen Virol. 2015 Aug;96(Pt 8):2036–49. doi: 10.1099/vir.0.000143 (PMC4681059; doi:10.1099/vir.0.000143)
Supplement: Supplementary file 1 — Supplementary Data [file vir-96-08-2036-s001.pdf]

Title: The 20 amino acids at the C-terminus of PA-X are associated with increased influenza A virus replication and pathogenicity

Huijie Gao[1#], Honglei Sun [1#], Jiao Hu [2], Lu Qi [1], Jinliang Wang [1], Xin Xiong[1], Yu Wang [1], Qiming He [1], Yang Lin [1], Weili Kong [1], Lai-Giea Seng [3], Juan Pu [1], Kin-Chow Chang [3], Xiufan Liu[2], Jinhua Liu [1], and Yipeng Sun [1,*]

Author Affiliation:

[1] Key Laboratory of Animal Epidemiology and Zoonosis, Ministry of Agriculture, College of Veterinary Medicine and State Key Laboratory of Agrobiotechnology, China Agricultural University, Beijing, China

[2] Animal Infectious Disease Laboratory, School of Veterinary Medicine, Yangzhou University, Yangzhou, Jiangsu Province, China

[3] School of Veterinary Medicine and Science, University of Nottingham, Sutton Bonington Campus, United Kingdom

*Corresponding author. Key Laboratory of Animal Epidemiology and Zoonosis, Ministry of Agriculture, College of Veterinary Medicine, China Agricultural University, No. 2 Yuanmingyuan West Road, Beijing 100193, China.

Tel: +86-10-62732982; Fax: +86-10-62732982; E-mail: sypcau@163.com

[#] Huijie Gao and Honglei Sun contributed equally to this work.

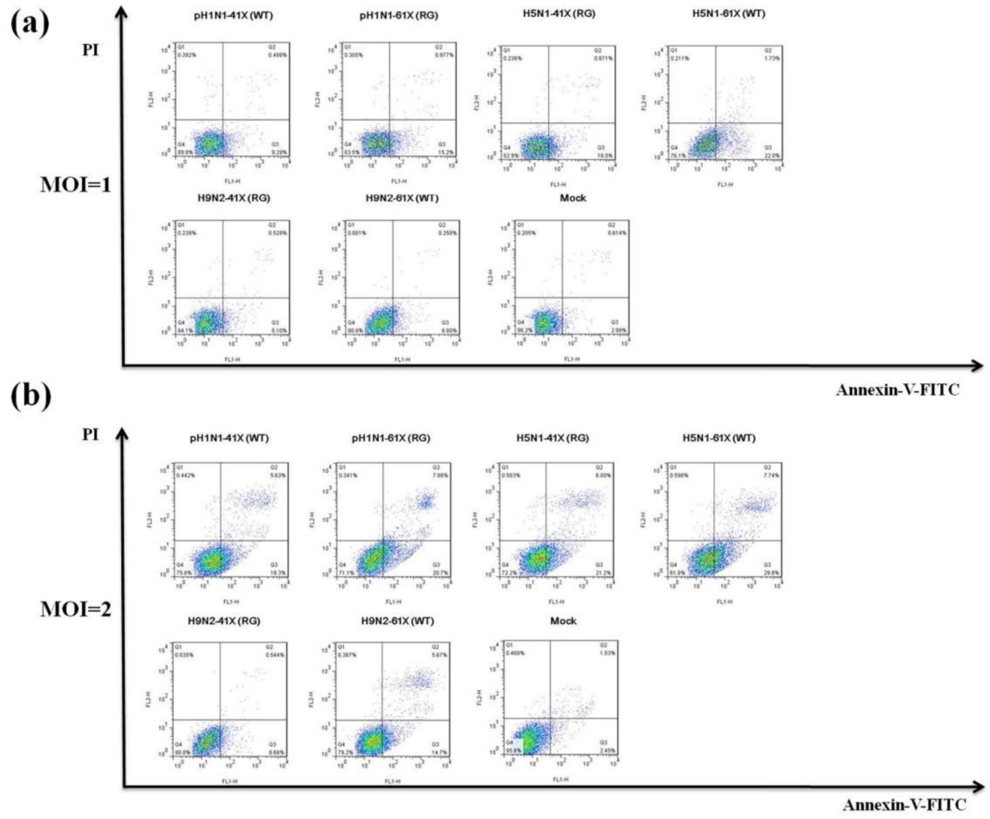

Figure. S1. Apoptosis of PA-X mutant viruses in A549 cells. Relative induction of apoptosis and necrosis as determined by the detection of annexin+, PI+ and annexin+PI+ in A549 cells infected with the panel of indicated viruses at a 1.0 (a) and 2.0 (b) MOI for 12 hours. Representative dual-labeled quadrants of bivariant fluorescence dot plots show the relative induction of apoptosis (annexin+) and necrosis (PI+) in infected cells. Apoptotic cells that were positive for annexin V but not PI were identified in the right lower quadrant, and those positive for PI but not annexin V were identified in the left upper quadrant. Percentages shown are proportions of apoptotic cells. Mock, uninfected control cells.
